# Gut-Muscle Axis Exists and May Affect Skeletal Muscle Adaptation to Training

**DOI:** 10.3390/nu12051451

**Published:** 2020-05-18

**Authors:** Katarzyna Przewłócka, Marcin Folwarski, Karolina Kaźmierczak-Siedlecka, Karolina Skonieczna-Żydecka, Jan Jacek Kaczor

**Affiliations:** 1 Department of Bioenergetics and Physiology of Exercise, Medical University of Gdańsk, 80-210 Gdańsk, Poland; k.przewlocka@gumed.edu.pl; 2 Departmentof Clinical Nutrition and Dietetics, Medical University of Gdansk, 80-210 Gdańsk, Poland; marcinfol@gumed.edu.pl; 3 Department of Surgical Oncology, Medical University of Gdansk, 80-210 Gdańsk, Poland; leokadia@gumed.edu.pl; 4 Department of Human Nutrition and Metabolomics, Pomeranian Medical University, 70-204 Szczecin, Poland; karzyd@pum.edu.pl

**Keywords:** gut microbiota, athletes, muscle functions, gut-muscle axis

## Abstract

Excessive training may limit physiological muscle adaptation through chronic oxidative stress and inflammation. Improper diet and overtraining may also disrupt intestinal homeostasis and in consequence enhance inflammation. Altogether, these factors may lead to an imbalance in the gut ecosystem, causing dysregulation of the immune system. Therefore, it seems to be important to optimize the intestinal microbiota composition, which is able to modulate the immune system and reduce oxidative stress. Moreover, the optimal intestinal microbiota composition may have an impact on muscle protein synthesis and mitochondrial biogenesis and function, as well as muscle glycogen storage. Aproperly balanced microbiome may also reduce inflammatory markers and reactive oxygen species production, which may further attenuate macromolecules damage. Consequently, supplementation with probiotics may have some beneficial effect on aerobic and anaerobic performance. The phenomenon of gut-muscle axis should be continuously explored to function maintenance, not only in athletes.

## 1. Introduction

The intestinal microbiota consists of microorganisms inhabiting the gastrointestinal tract, with the estimated number exceeding 10^14^ cells. The genome size of microbiota exceeds the human genome by 150 times, which encompasses around 10 times more bacterial cells than all human cells and over [1,2]. The biodiversity and overall composition of the gut microbiota plays a crucial role in maintaining normal homeostasis within the human body. Bacteria are the most abundant population of the gut microbiota, with more than 1000 different bacterial species being observed. The human gut microbiota consists mainly of four phyla: *Firmicutes*, *Bacteroidetes*, *Proteobacteria*, and *Actinobacteria* [2]. An imbalance among these phyla may alter the microecological environment of the gastrointestinal tractand contribute to the development of various diseases. Intestinal bacteria are involved in many functions and have been shown toimpact the nutritional status of the host, metabolic functions, and maturation of the immune system, as well as epithelial cell maturation. Moreover, these bacteria protect against pathogens and may influence brain function [3,4]. Additionally, the composition of gut microbiota varies individually and may be modified by several factors, such as: genetic background (but to a smaller extent), age, sex, place of residence, and drug administration [5,6]. However, diet and the level of physical activity are the main determinates for altering the biodiversity or changing the levels of a specific bacterial species within an established gut microbiota [7].

Moderate physical activity has a multidirectional and beneficial effect on the human body. Training stimulation causes physiological and metabolic adaptations. Main changes in the skeletal muscles include the increase in mitochondrial biogenesis and enhancing their function, concentration of the substrate transporting proteins, activity of the enzymes involved in metabolic pathways, and glycogen storage in the muscle [8]. As a result of regular exercise, muscle protein synthesis is intensified with changes that vary based on the intensity of the training. It is regulated by physical and chemical mechanisms [9]. In brief, the signalling pathways include short-term alterations to protein turnover and genes expression, as well as long-term changes to metabolism within the cells. Additionally, the activation of the mammalian target of rapamycin kinase (mTOR) plays a crucial role for increasing muscle protein synthesis, through its phosphorylation of initiating substrates and its promotion of translational signalling for anabolism [9]. However, it should be noted that excessive exercise may limit muscle building and cause a net loss of muscle mass via promoting inflammation and nutrient restriction, as well as oxidative and metabolic stress. In this scenario, excessive exercise may lead to the activation of the muscle atrophy pathways, increasing the levels of nuclear factor kappa B (NF-κB) or Forkhead box O (FOXO) in their phosphorylated forms [10,11].

Although multiple studies have illustrated that moderate physical activity has a beneficial effect on the gut microbiota, it is unclear if the gut microbiota influences muscle adaptation to extensive training. A recent study showed that excessive exercise among professional athletes may disturb the homeostasis of the gut microbiota [12]. Specifically, high volume training was associated with increased muscle requirements for oxygen and nutrients. Furthermore, long-term deterioration of the intestinal blood perfusion has been shown to cause temporary ischemia, leading to the dysfunction of the mucous membrane and increase of intestinal permeability [13]. As a result, substantial changes of the microbiota profile were observed via promoting a bloom of opportunistic pathogens and their associated toxins. Consequently, it could lead to the translocation of pathogens and bacterial toxins into the bloodstream, resulting in the activation of local and systemic inflammatory pathways [14]. From these studies, it is clear that the maintenance of a healthy microbiome, within the gut, does influence muscle adaptation to training. Specifically, the microbiota may have an indirect role through its modulation of inflammatory pathways and anabolic and catabolic processes, as well as the regulation of nutrient availability and metabolite production.

## 2. The Link between Diet, Physical Activity, and Microbiota

The composition and quality of diet significantly affect the exercise capacity of athletes. Adequate energy, macro- and micronutrients intake are essential to optimize protein synthesis, increase of energy reserves during exercise, improve regeneration after training, and reduce the risk of injury. Insufficient energy intake has multiple negative consequences, referred to as relative energy deficiency in sports (RED-S) [15]. It may impair sport performance through endocrine or immune system disorder, insufficient muscle glycogen storage and microbiome imbalance [16]. As such, the intake of carbohydrates, fats, and proteins, as well as the preservation of a healthy gut microbiome, are essential for maintaining an athlete’s exercise capacity. 

As the main indirect energy substrate for skeletal muslces, carbohydrates and their storage as glycogen, have a clear role in proper muscle function during both aerobic and anaerobic exercise. Specifically, an individual’s ability to store carbohydrates as glycogen has been shown to affect mitochondria biogenesis and function as well as acting as a specific regulator for signalling pathway involved in training tolerance [17,18]. Intestinal bacteria also have a role for mainitaining exercise capacity through regulation of carbohydrates. They promote the colon fermentation of carbohydrates to produce short-chain fatty acids (SCFAs) from undigested fragments. SCFAs are characterized by a multiple of positive effects on the host organism, including the improvement of metabolic function and enhancement of intestinal epithelial membrane [19,20]. Moreover, diets that reduce carbohydrate intake are linked to negative effects on exercise capactity, due to the association with increased fat consumption.

A low-carbohydrate diet with a high fat content impairs exercise economics and inhibits the growth of workout-induced aerobic fitness, in contrast to the high-carbohydrate diet [21]. Additionally, excessive fat intake may also significantly affect the composition of the intestinal microbiota limiting substrates to SCFAs production. Animal studies have shown an increase in the number of bacteria that induce pro-inflammatory cytokines synthesis elevates the content of plasma lipopolysaccharide (LPS) as well as enhancing NF-kB expression, linked to turning on the genes of pro-inflammatory character [22]. The high-fat diet also reduces the diversity of bacterial strains and the abundance of *Bacteroidetes*, promoting the growth of *Firmicutes* and *Proteobacteria* [23]. Furthermore, an increased amount of sulfate-reducing bacteria have also been demonstrated. These bacteria may produce sulfides, which lead to the reduction of disulfide bonds in the mucus and the breakdown of gel-forming polymer protein networks MUC2 secreted by goblet cells. These alterations play pivotal role in mucosal regeneration and mucus layer stability. An impaired mucosal barrier may exacerbate intestinal mucosa inflammation and promote inflammatory diseases [24]. All these observations were reported in the case of high-fat diet, containing mainly saturated fats and processed food. However, in the case of omega-3 acids and conjugated linoleic acid (CLA) unfavorable changes were not found. Their consumption increased butyrate synthesis and the *Bacteroidetes/Firmicutes* ratio [25]. 

Adequate protein intake is essential for maximizing muscle adaptation to the training processes, conducive to hypertrophy and muscular strength [26]. However, excessive protein intake causes an increase in the number of protein fermenting bacteria such as *Clostridium*, *Desulfovibrio*, *Peptostreptococcus*, *Acidaminococcus*, *Veillonella*, *Propionibacterium*, *Bacillus*, *Bacteroides*, *Staphylococcus*, and other species of the *Proteobacteria* family [27]. It was also associated with reducing the number of carbohydrate fermenting bacteria such as *Bacteroides*, *Lactobacillus*, *Bifidobacterium*, *Prevotella*, *Ruminococcus*, *Roseburia*, and *Faecalibacterium* [28,29]. The fermentation of undigested protein residues in the colon, accompanied by the production of by-products, such as ammonia, biogenic amines, indole compounds, and phenols, have a potentially harmful effect on the intestine, metabolism, immunological, and neurological functions. These compounds may exacerbate the inflammatory response, increase tissue permeability, and intensify gastrointestinal symptoms [30]. It appears that protein overconsumption may be offset by higher carbohydrates intake, especially indigestible polysaccharides, which are the preferred substrate for intestinal bacteria [30].

Moderate training has a beneficial effect on the diversity of bacterial species inhabiting the gastrointestinal tract. The microbiome of various athletes has been correlated with high diversity and increased levels of bacterial genes involved in protein and carbohydrate metabolism and SCFAs production [31,32]. In addition, research conducted on cyclists showed that higher activity for carbohydrate metabolizing bacteria correlated with the frequency of exercise. Moreover, increasing the number of *Prevotella* was demonstrated to positively affect amino acid metabolic pathways, such as lysine biosynthesis, the metabolism of alanine, aspartate, and glutamate, d-glutamine and d-glutamate, as well as carbohydrate metabolism. In high-performance athletes, a larger share of methane-producing bacteria from the *Methanobrevibacter Smithii* family was also associated with an excessive production of energy and carbohydrate metabolism [33]. The study conducted by Durk et al. also found a positive link between the level of training expressed by maximal oxygen uptake (VO_2max_) and the *Firmicutes/Bacteroidetes* ratio [34]. From an inflammatory standpoint, training-induced changes in intestinal microbiome composition seem to be beneficial to host health. Regular exercise may also support brain functions via enhancing the neuroprotective effect. As a result of training, an increase of gene expression of the kynurenine aminotransferases occurred, which are responsible for the conversion of the toxic metabolite tryptophan—kynurenine to the neuroprotective kynurenic acid. Inflammatory cytokines such as tumor necrosis factor α (TNF-α) have also been shown to promote the degradation of kynurenine to toxic quinolinic acid [35]. In addition, it seems that the optimal intestinal microbiota composition may have a positive effect on the brain function and preventing depression, by modulating inflammation and affecting tryptophan metabolism. All of these may indirectly affect the quality of physical training [36].

As stated previously, excessive training may introduce microecological imbalances via intestinal ischemia, increased intestinal barrier permeability, and elevated oxidative stress. This leads to the exacerbation of inflammatory responses, and consequently, to increased catabolism along with muscle function deterioration. Adverse effects may also result from an increase ofa number of potentially harmful bacteria, such as *Peptostreptococcus*, *Staphylococcus*, *Peptoniphilus*, *Acidaminococcus*, and *Fusobacterium*, and a decrease of anti-inflammatory species including *Bacteroides*, *Faecalibacterium*, *Collinsella* and *Roseburia*. This was clearly shown in the study conducted by Karl et al. that analyzed stool samples of soldiers under prolonged physiological stress [37]. They showed an indirect relationship between intestinal microbiota composition, lifestyle, and skeletal muscle function. It supports the hypothesis of the gut-muscle axis and the necessity of targeted therapy for the microbiota athletes.

During physical training, there is an overproduction of reactive oxygen species (ROS), as a result of increased skeletal muscle effort. ROS generation causes lipid and protein peroxidation, muscle cell membranes components disruption, which all together consequently disturb muscle function [38]. Therefore, both training overload and lack of physical activity, as well as immobilization, raise oxidative stress [39,40]. On the other hand, regular training leads to the adaptation of antioxidant enzymes, increasing the activity of superoxide dismutase (SOD), catalase (CAT), and glutathione peroxidase (GPx). It also reduces the damage caused by free radicals and increases the antioxidant potential and the activity of enzymes responsible for repairing damages caused by ROS [41]. These findings were supported by studies conducted by Maleki et al. They demonstrated that higher SOD and CAT activity, together with lower ROS levels occurred in the semen of participants performing recreational training compared to inactive participants or professional athletes [42]. Similar observations were made by Brinkmann et al., who reported that moderate intensity exercises induced higher SOD and GPx activity in the skeletal muscle [43]. In addition, ROS production has been shown to have a positive effect on aerobic potential by activating PGC-1α proteins. It leads to the increase of mitochondrial biogenesis and consequently the improvement of the aerobic capacity [44]. Previous studies have shown that ROS regulate muscle protein synthesis by affecting mitogen-activated protein-kinase (MAPK) activity, which supports the pro-anabolic insulin-like growth factor 1 (IGF-1) [45]. Recently, it has also been suggested that excessive supplementation of antioxidants can reduce cytochrome c oxidase and citrate synthase content, which impairs the electron transport chain (ETC) functions [44].

**Figure 1:**
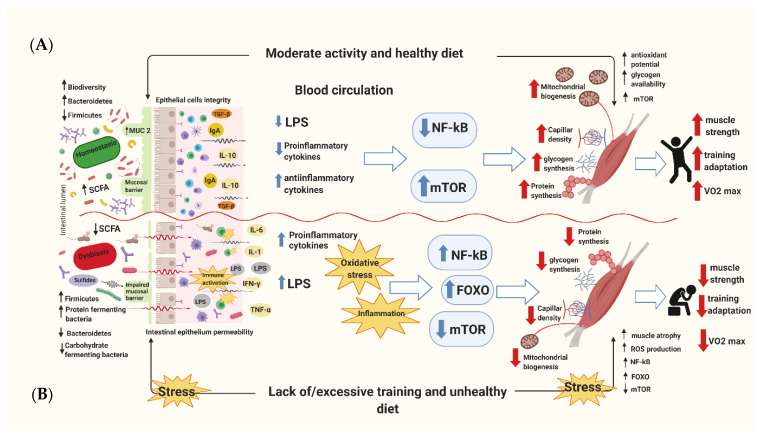
The schematic diagram of combined healthy/unhealthy diet and exercise/lack of exercise action on human skeletal muscle. (**A**) A properly balanced diet and systematic moderate exercise show both direct and indirect effects to benefit skeletal muscle function by reducing oxidative stress and inflammation status. As a result, this shifts to the higher muscle glycogen storage and increased mitochondrial biogenesis and function, as well as the predominance of anabolic signaling pathways, which increasethe aerobic exercise capacity. (**B**) The opposite effects are observed in the case of an inadequately balanced diet and insufficient or excessive physical effort. This leads to an increase in inflammatory and oxidative stress markers, a decrease in the ability to store muscle glycogen and a reduction of mitochondria function, as well as muscle atrophy and the higher accumulation of body fat.

The intestinal microbiome may also contribute to oxidative stress reduction. Some bacterial strains have antioxidant properties through various mechanisms. These include the expression of antioxidant enzymes, modulation of inflammation caused by pro-inflammatory cytokines or presence of pathogens, and metabolism regulation through greater absorption of antioxidants [46]. Specifically, some studies have shown that bacterial species such as *Lactobacillus plantarum*, *Lactobacillus gasseri*, *Lactobacillus fermentum*, *Lactococcus Lactis* and *Streptococcus thermophilus* are able to increase SOD activity [47]. Additionally, *Lactobacillus*, *Lactococcus*, and *Bifidobacterium* genera have all been shown to elevate intestinal glutathione (GSH) levels, which plays a crucial role in scavenging of the hydroxyl radical (OH*) [47]. Similarly, animal studies have demonstrated that individuals whose microbiota was richer in *Escherichia coli* and *Enterococci*, while being poorer in *Lactobacilli*, had higher susceptibility to oxidative stress [48]. Martatelli et al. conducted a trial with athletes, showing that a *Lactobacillus rhamnosus* and *Lactobacillus paracasei* probiotic species supplementation increased plasma antioxidant levels and neutralized ROS generation as a response to high-intensity exercise. Probiotic supplementation was also associated with lower plasma reactive metabolite levels and higher plasma biological antioxidant potential, after afour-week intensive physical training period [46]. Overall, these findings clearly support the essential need to balance a proper diet, an adequate exercise regime, and a healthy microbiome to promote higher glycogen storage to increase mitochondrial function and muscle building. On the other hand, an inadequately balanced diet and an insufficient or excessive training regime, as well as a dysfunctional microbiome, are all associated with increased inflammation, oxidative stress, a reduction in mitochondrial function, and the potential for muscle atrophy (Figure 1).

## 3. The Effect of Microbiota on Anabolic and Catabolic Processes

The intestinal microbiome may affect the metabolism of human skeletal muscles through several pathways. Evidence regarding the relationship between microbiota composition and muscle function have been described in the pathogenesis of age-related sarcopenia. It was noted that muscle atrophy correlates with a decrease in the number of species sending anti-inflammatory and pro-anabolic mediators. Sarcopenia is associated with a reduction of muscle capillaries and a decrease of insulin sensitivity and inflammation severity, leading to declined mitochondrial biogenesis and function as well as protein synthesis disruption [49].

Sarcopenia and systemic weakness among the elderly have been correlated with intestinal dysbiosis, contributing to increased intestinal barrier permeability, elevated blood LPS levels, activation of the immune system, and areduction of insulin sensitivity [50]. Moreover, animal studies clearly emphasize the reduction of muscle atrophy markers (Atrogin-1, MuRF1, LC3 protein, Cathepsin L) in mice supplemented with *Lactobacillus* strains as well as an increase of muscle mass and strength in mice supplemented with *Lactobacillus plantarum* [51,52]. In addition, Buigues et al. demonstrated 13-week multistrain *Lactobacillus* and *Bifidobacterium* probiotic mixture supplementation enhanced endurance and muscular strength in older individuals. The study showed that older patients who received fructooligosaccharides and inulin experienced a significant improvement in hand grip strength andself-reported exhaustion level [53].

The lack of homeostasis was associated with an increased abundance of endotoxic gram-negative bacteria responsible for thesystemic inflammation via LPS. It has also been noticed that *Escherichia/Shigella*, *Klebsiella*, and *Citrobacter* species significantly contribute to the LPS pool [54]. Elevated serum LPS levels have been correlated with the increased *Firmicutes/Bacteroidetes* ratio [55]. Consequently, the presence of LPS in bacterial cell walls causes binding of the lipid A to the surface of immune cells receptors, containing TLR4 and bone marrow differentiation factor 2 (DM2). LPS are recognized by TLR4 in combination with CD14 and DM2, and therefore, may induce the NF-κB activation, which plays a key role in the production of pro-inflammatory cytokines [14,56]. Additionally, elevated LPS levels have been connected with intestinal homeostasis disruption and correlated with an increase in blood intestinal permeability markers such as zonulin and fatty acid-binding protein 2 (FABP2) [57]. This augmented permeability of the intestinal epithelium is associated with bacterial translocation from the intestinal lumen to the lamina propria, activating the immune system and promoting inflammation. However, it should be noted that *Actinobacteria* genus bacteria, such as *Bifidobacterium* or *Collinsella*, have been shown to have anti-inflammatory and immunomodulatory properties that can support intestinal epithelial function. Therefore, probiotics containing *Bifidobacterium* strains may reduce the inflammatory response caused by physical stress [58].

SCFA produced by intestinal bacteria have also been demonstrated to have a positive effect on the integrity of the intestinal barrier, protecting it against inflammation. Specifically, the *Candida Albicans* genus has been shown to be engagedin pro-inflammatory TNF-α induction [59]. Dysbiosis has often been accompanied by an increase in the amount ofgram-negative bacteria that have endotoxic properties and upregulate pro-inflammatory cyotkines like IL-6 [60]. Elevated intestinal permeability level and associated passage of pathogens into the bloodstream induce IL-1, TNF-α, and interferon gamma (IFN-γ) secretion, causing a pro-inflammatory effect [61]. The intestinal microbiota composition can also affect the inflammatory suppression by stimulating secretion of anti-inflammatory cytokines, such as transforming growth factor (TGF-β) and IL-10. It has been proven that *Bacteroides fragilis* bacteria were able to suppress the expansion of Th17 lymphocyte by producing IL-10 via the TLR2 [62]. *Lactobacillus* and *Bifidobacterium* families are associated with inflammation reduction, by affecting the secretion of anti-inflammatory cytokines such as IL-10, TGF-β, and tryptophan-2,3-Dioxygenase (IDO), causing Treg stimulation as well as Th1, Th2, and helper lymphocytes Th17 inhibition [63]. Dysbiosis through the loss of immune tolerance impairs epithelial and intestinal barrier functions. Consequently, this disturbs the balance between pro- Th17 and anti-inflammatory Treg lymphocytes.

Muscle protein synthesis and training adaptation may be limited under chronic inflammation. Notably, satellite cells located between the basal lamina and plasma membrane for muscle fibers play a key role during regeneration and muscle growth [64]. Muscle fiber synthesis and breakdown are under the control of many crossing signaling pathways, which determinate anabolic and catabolic processes. Two E3 ubiquitin ligases, belonging to the ubiquitin-proteasome system, are mainly involved in muscle protein degradation: Atrogin-1 and Muscle RING finger protein (MuRF1). The increase of their transcription activity is regulated by the NF-kB nuclear factor and phosphorylated FOXO proteins. Therefore, the inhibition of these signalling pathways are associated with protection against skeletal muscles atrophy [65,66]. The secretion of pro-inflammatory cytokines also activates NF-kB, contributing to skeletal muscle loss. This is mainly mediated by TNF-α, capable of activating IκB (IKKβ), whose active form may phosphorylate IκB proteins, thereby triggering the NF-kB signaling and changing gene transcription towards catabolism [67].

Myofibrillar protein synthesis is dependent on extracellular signals that activate intracellular molecular pathways. It seems that mTOR plays a crucial role in the process of muscle protein synthesis. Its activation leads to the intensification of anabolic processes, through the integration of signalling pathways that increase translational efficiency and the phosphorylation of initiate substrates [9]. mTOR phosphorylation may be stimulated by either training or nutritional support. Mechanical contractions during resistance training results in the release of IGF-1 from skeletal muscles, capable of mTOR activation. Protein or amino acid intake also contributes to enhanced mTOR signalling, demonstrating a synergistic effect to the exercise stimulus [68]. IGF-1 secreted into the extracellular matrix is bound by specialized IGF-binding proteins (IGFBP), enabling the activation of specific receptors that process the anabolic signal [69].

Physical training leads to a decrease of adenosine triphosphate ATP level and disturbances in the ATP/AMP (adenosine monophosphate) ratio, causing an energy stress occurrence. The higher concentration of AMP stimulates AMP-activated protein kinase (AMPK) to equalize energy resources by initiating catabolic processes. AMPK promotes aerobic and anaerobic energy production, inhibits glycogen as well as cholesterol synthesis, and induces mitochondrial biogenesis through PGC-1α expression [70]. The biological role of AMPK also controls the circulation of cellular components by reducing mTOR activity and promoting protein breakdown. The elevated AMPK level positively correlates with the increase of FOXO protein activation [71]. The stress response causes FOXO proteins phosphorylation, which intensifies autophagy genes transcription, contributing to the protein breakdown (mainly FOXO3). However, regular exercise does induce autophagy, which isa necessary step prior to muscle fiber rebuilding. It is clear that elevated autophagy is associated with impairments in muscle growth and function [70,72].

Excessive training load and insufficient regeneration periods may cause exhaustion and a temporary weakening of sport performance. Therefore, appropriate regeneration after exercise is an important element in training adaptation [73]. Physical exercise-induced tissue damage is a physiological part of the adaptation process; however, chronic training overload and insufficient regeneration may adversely affect the athlete’s well-being and sport capabilities [74]. Specifically, the tissue damage caused by excessive training may result in an acute and local inflammatory response, consisting of cytokines overproduction, mainly interleukin-1b (IL-1b), TNF-a, interleukin-8 (IL-8), and interleukin-6 (IL-6) aimed at rebuilding the damaged structures and promoting muscle adaptations. As a result, there is an activation of circulating monocytes, capable of pro-inflammatory cytokines induction and causing systemic inflammation [75,76], leading to insulin resistance, endoplasmic reticulum stress, and, as a result, muscular atrophy [73]. Moreover, ROS generation may disrupt protein synthesis, promote inflammatory response, and reduce the efficiency of post-workout regeneration processes [77].

Jäger et al. have demonstrated the beneficial effect of using *Streptococcus thermophilus* FP4 and *Bifidobacterium breve* BR03 strains supplementation, to regulate the inflammation state and enhance muscle training adaptation. The study showed that 21 days of probiotic supplementation period decreased blood IL-6 level, 48 h after eccentric exercise in 15 trained men. It also reduced the movement limitations caused by training, contributing to a shortening of the regeneration period [78]. The positive effects on inflammation parameters and muscle functions were also demonstrated by Wen-Ching et al. They have noticed that long-term *Lactobacillus plantarum* PS128 supplementation in triathletes resulted in the reduction of plasma creatine kinse (CK) level. Additionally, other significant improvements were found across various markers of inflammation and oxidative stress during the regeneration phase. These improvments manifested in myeloperoxidase (MPO) and IL-10 elevation as well as a TNF-α, IFN-γ, IL-6, and IL-8 decrease [79]. The effectiveness of probiotic supplementation was also demonstrated by Townsend et al., who showed that 12 weeks of *Bacillus subtilis* DE111 treatment reduced TNF-α levels, without altering other inflammation parameters [80]. Another study, conducted by Roberts et al., clearly displayed the positive effect of using a multi-strain probiotic (*Lactobacillus acidophilus* CUL-60, *Lactobacillus acidophilus* CUL-21, *Bifidobacterium bifidum CUL*-20, and *Bifidobacterium animalis*) for 12weeks on the intestinal permeability of triathlonists. The probiotic combined with fructooligosaccharides and α-lipolic acid supplementation was associated with a reduction in blood endotoxins level compared to the control group [81].

## 4. Bacterial Products and Their Effect on Muscle Function

Intestinal bacteria can affect the human body by producing a variety of biologically active metabolites. One of the best-known bacterial metabolites are SCFAs. It was considered that SCFAs may be provide the source of up to 10% of total daily energy demands [82]. Butyrate, acetate and propionate are the most known SCFAs, representing as much as 95% of all SCFAs.

It seems that butyrate plays a key role in regulating cell growth and differentiation [83]. The *Roseburia*, *Clostridia*, and *Eubacteria* genus are main butyrate producers [4]. There are a number of anti-inflammatory properties associated with butyrate, such as enhancing intestinal barrier integrity, promoting antimicrobial peptides secretion, Treg lymphocyte activation, regulation of neutrophil migration, TLR silencing, decrease of pro-inflammatory cytokines production, and lymphocyte or granulocyte activity suppressing. Additoinally, butyrate has been shown to suppress the inflammatory response by altering NF-kB and protein kinase B (AKT) signalling [84] and antagonizing LPS. Additionally, it reduces intestinal permeability, improves tissues’ insulin sensitivity, increases lipolysis, and stimulates skeletal muscle glucose uptake [49].

Similar anti-inflammatory properties have been observed for acetate. It affects glucagon-like peptide-1 (GLP-1) and YY peptide secretion, resulting in appetite inhibition, lipolysis, and energy expenditure increase. Moreover, acetate has a beneficial effect on skeletal muscle by stimulating glucose uptake and increasing insulin sensitivity [85]. Propionate and butyrate regulate the secretion of intestinal hormones, improving insulin sensitivity and affecting glucose metabolism [86], becoming a gluconeogenesis precursor and lipogenesis inhibitor [87].

The direct relationship between SCFA and skeletal muscles is mediated by muscular AMP kinase and the deposition of proteins in skeletal muscle tissue. SCFA activate AMPK by increasing the AMP/ATP ratio or via the Ffar2-leptin pathway, but the exact mechanism is not known [88]. Intestinal bacteria may produce secondary bile acids, having antibacterial activity. It has been shown that microbiota may affect the liver and the skeletal muscle receptors, modulating the activity of the farnesoid X receptor (FXR) [89]. This receptor plays an important role in energy metabolic pathways, lipoprotein and glucose turnover. Intestinal microbiota, by alleviating FXR inhibition may contribute to the metabolic balance maintenance and myocyte anabolism. In addition, bile salts may be transformed into immunomodulatory and anti-inflammatory compounds in the intestine [90,91].

## 5. Microbiome and the Availability of Nutrients

Intestinal microbiota affects the availability and profile of amino acids by participating in their digestion and absorption. Notably, *Fusobacterium*, *Bacteroides*, *Veillonella*, *Megasphaera elsdenii*, and *Selenomonas ruminantium* are all involved in proteolysis, increasing the disposal of amino acids [92]. In addition, some bacterial species such as *Streptococcus bovis*, *Selenomonas ruminantium*, and *Prevotella bryantii*, in the presence of physiological peptide concentrations are involved in *de novo* biosynthesis of amino acids [93]. Intestinal bacteria are crucial for tryptophan metabolism by its direct consumption, thus limiting the availability to the host organism [36]. On the other hand, the intestinal microbiota composition is a key determinant of tryptophan metabolites level in the circulation and serotonin (5-HT) in the brain [92] consequently negatively affects muscle training adaptation.

Another crucial role of microbiota is in the production of vitamins, such as folates, riboflavin (B_2_), cobalamin (B_12_), and vitamin K. Vitamins B are necessary for myocytes anabolic processes through various pathways and a several of metabolic functions, including DNA replication and repair and nucleotide and amino acid synthesis, as well as oxidative stress regulation. *Bifidobacterium longum*, *Bifidobacterium bifidum*, and *Lactobacillus reuteri* are all involved in vitamin synthesis [94]. Intestinal bacteria are also able to metabolize polyphenols, but their efficiency may decrease under unfavourable conditions within the gut. Polyphenols have antioxidant and anti-inflammatory properties and also contribute to mitochondrial biogenesis and function [95].

Lactate utilizing bacteria seem to have an important role for athletic exercise capacity. Lactate is able to penetrate from the serum into the intestinal lumen where it is converted to SCFAs, mainly propionate. Then, the SCFAs enter directly into the circulation where through the Cori cycle transformations, become an additional energy source [96]. Recent studies conducted by Scheiman et al. have shown the important role of *Veillonella atypica* genus, whose only source of carbon is lactate. The number of these bacterial genera was elevated in the intestines of high-performance athletes. It has also been shown that transplantation of *Veillonella atypica* genus into mice was associated with a significant running time improvement. Therefore, it has been reported that the modulation of enzymes and conversion of lactate to propionate has a role in improving atheletic performance [97]. Lastly, animal models have illustrated the role of SCFA (mainly propionate) in maximizing oxygen uptake and elevating heart rate, while in humans it may cause a resting energy expenditure increase [98,99].

## 6. Glucose Metabolism

In the light of the current knowledge, the expression of intestinal receptors Gpr41 and Sglt1, involved in glucose transport and energy balance, is associated with an increase in skeletal muscle oxygen metabolism. Bacterial SCFA are able to activate Gpr41 receptors, affecting the endocrine pathway to release the glucagon-like peptide 1 (GLP-1), stimulating insulin secretion [100,101]. A similar mechanism is observed in the case of the sodium glucose co-transporter Sglt1, responsible for glucose homeostasis. Nay et al. have reported that antibiotic-treated mice showed reduced expression of Gpr1 and Sglt1 genes, which is correlated with muscle glycogen content reduction compared to the control group [101].

The intestinal dysbiosis, often caused by antibiotic therapy, contributes to alterations in SCFAs and bile acids (BA) synthesis, which was shown in Zarrinpar et al. The limitation of butyrate, the main energy source for enterocytes, causes glucose compensation. Consequently, this translates into low serum glucose levels as well as insulin sensitivity and increased hepatic gluconeogenesis [102]. It has also been reported that intestinal dysbiosis may reduce skeletal muscle glucose availability, resulting in the reduction of glycogen storage. The glycogen content in muscles is a key factor determining an athletes’ aerobic energy metabolism. Glycogen level disturbances may cause muscle strength and function deterioration, leading to bioenergetic metabolism impairment [18]. This concept was supported by another study, which correlated between intestinal microbiota composition and muscle glycogen content. Germ-free mice were shown to have lower muscle glycogen levels compared to individuals with normal microbiome composition [101]. This data demonstrates the important role of microbiota in skeletal muscle function by improving the availability of energy substrates such as glucose.

## 7. The Interaction between Microbiota and Mitochondrial Function

Intestinal microbiota may affect mitochondrial functions in various ways. LPS, produced mainly by pathogenic bacteria, activates NF-kB signalling and an inflammatory response, through TLRs, resulting in pro-inflammatory cytokine production. TLR activation indirectly increases ETC activation, leading to mitochondrial ROS generation [103]. It has been noted that the growth of pathogenic *Listeria monocytogenes* species contributes to mitochondrial networks fragmentation, disrupting their function [104]. Other intestinal bacteria such as *Mycobacterium tuberculosis* and *Ehrlichia chaffeensis* have been shown to reduce ROS generation, by inhibiting LPS-initiated pathways or by increasing SOD activity [103].

Moreover, it has also been reported that amino acid-reducing bacteria, e.g., *Escherichia coli* and *Salmonella*, are capable of hydrogen sulfide (H_2_S) production. In large quantities, H_2_S inhibits the mitochondrial ETC, by lowering cytochrome c oxidase activity [103]. Other bacterial metabolites, such as SCFAs, may contribute to the regulation of aerobic energy metabolism in the skeletal muslces. This mainly occurs through butyrate and its ability to enter the Krebs cycle to increase its efficiency [105]. However, recent data has suggested that isovanillic acid 3-O-suflate (IVAS) may also have a positive effect on the glucose absorption and metabolism in human cells. IVAS was shown to increase glucose transport in a dose-dependent manner by activating GLUT-4 and GLUT-1, phosphatidylinositol 3-kinase (PI3K) and AKT phosphorylation [106]. PI3K seems to be crucial for muscle metabolism and mitochondrial homeostasis by modulating insulin sensitivity [107].

## 8. Microbial Modulation of Neuroactive Molecules

Recently, multiple studies have supported the existence of a gut-brain axis (GBA) that enables bidirectional communication between these two organs. Its signalling pathways consist mainly of afferent and efferent neurons proceeding through the sympathetic and parasympathetic fibers of the autonomic nervous system (ANS). Using that bidirectional cross-talk, intestinal signals are able to affect the brain function, regulating mood or even reflex activity. Similarly, the central nervous system may alter gastrointestinal’s (GI) track motility and acid secretion in the stomach and control the defecation process [108,109].

It has been established that the gut microbiota plays a crucial role in gut-brain communication by generating some neuroactive molecules. For example, strains of *Lactobacillus* genus were demonstrated to produce γ-aminobutyricacid (GABA), an important inhibitory transmitter in the brain. Similarly, other bacterial species were shown to be capable of noradrenaline (e.g., *Bacillus mycoides*, *Bacillus subtilis*), dopamine (e.g., *Bacillus cereus*, *Bacillus mycoides*, *Bacillus subtilis*), and serotonin (e.g., *Lactococcus lactis*, *Lactobacillus plantarum*, *Streptococcus thermophilus*) synthesis [108,110,111]. Therefore, it is clear that intestinal bacteria have the potential to alter neurotransmitter activity, thus interacting with the host nervous system to regulate mental health, and consequently, metabolism and exercise capacity.

Supporting these findings, a recent systemic review illustrated how moderate training contributes to the elevation of GABA level in the hypothalamus, which is associated with lowered resting blood pressure, heart rate, and sympathetic tone. In addition, dopamine was shown to be synthesized in the GI tract, during stressful situations. On the other hand, training overload was reported to cause muscle exhaustion and modifications in the CNS leading to mood disturbances, fatigue, insomnia, and depression. The central fatigue was associated with the elevation of 5-HT release and could lead to suboptimal physical performance. Consquently, this reduction in 5-HT levels in the brain could lead to the manifestation of mood disorders, depression, distorted cardiac function, and changes in blood pressure. Overall, gut microbiota was shown to facilitate the production and regulation of neurotransmitters and hormones, which consequently affected athletes well-being, mood, motivation, and subjective sense of regeneration [112].

Interestingly, the study conducted by Bravo et al. presented that chronic supplementation with *Lactobacillus rhamnosus* caused alternations in central GABA receptors expression, reducing stress-induced corticosterone (CORT) as well as anxiety- and depression-related behavior [113]. Furthermore, 5-HT levels were shown to be lower in the blood and colon of GF animals as compared to their typically colonized counterparts. It was suggested that this effect was dependent on bacterial molecules such as SCFA [114]. Additionally, Crumeyrolle-Arias et al. demonstrated the important role of gut bacteria in response to stress. GF mice exhibited higher serum CORT concentrations, elevated corticotropin-releasing factor mRNA expression in the hypothalamus and lower dopaminergic turnover rate in the hippocampus compared with specific-pathogen free mice. These changes suggest that the lack of the gut microbiota exacerbates stress response [115]. Furthermore, the chronic elevation in endogenous glucocorticoids levels may have decreased the rate of protein synthesis and increased proteolysis, to generate amino acids that serve as precursors for hepatic gluconeogenesis. However, in skeletal muscles, this may lead to the development of oxidative stress [116] and skeletal muscle atrophy, as well as muscle weakness [117,118]. Based on these data, we presumed that the gut microbial composition plays a crucial role in the development and function of an appropriate stress response via hypothalamus–pituitary–adrenocortical axis regulation, andas a consequence, exercise abilities in athletes.

## 9. Impact of the Microbiome on Exercise Capacity

Numerous researches have indicated the validity of intestinal microbiota-targeted strategies to improve training parameters and increase training capabilities, as presented in Table 1. The researches indicate the ability of intestinal microbiota to alleviate oxidative stress and exercise-induced inflammation [78,79,80,119,120]. A trial conducted by Jager et al. have shown that *Bacillus coagulans* GBI-30 probiotic supplementation improves the anaerobic capacity measured by the Wingate Test [121]. The positive properties of probiotic supplementation on post-workout regeneration have been presented by Carbuhn et al. as well as Huang et al., using *Bifidobacterium longum* 35624 [122] and *Lactobacillus plantarum* PS128 [79], respectively. In both study groups, athletes reported a feeling of faster recovery time in the probiotic group compared to the placebo groups.

Animal studies have also shown a positive probiotic effect on the aerobic fitness of athletes through extending the exercise to exhaustion time. Hsu et al. have observed muscle mass and endurance augmentation, as well as the antioxidant potential in mice with optimal intestinal microbiota composition [119]. These observations were consistent with the subsequent medical experiment carried out by Chen et al. [52]. Similar reports come from Scheiman et al., who demonstrated the bacterial role in lactate utilization, and thus in increasing exercise capacity [97].

## 10. Conclusions 

In the light of current knowledge, it seems that intestinal microbiota intervention may have beneficial effects on the human body, resulting in better athletic performance. Modulation of the immune response, oxidative stress, metabolic processes, and nutrients bioavailability are considered the main mechanism(s) by which the microbiota affects training adaptation. The microbiome may also have an impact on muscle protein synthesis and mitochondrial biogenesis and function, as well as muscle glycogen storage. Dysbiosis may reduce physiological adaptation, increase inflammatory markers and ROS generation as well as free radical macromolecules devastation, all contributing to skeletal muscle atrophy. On the other hand, numerous studies indicate the beneficial effect of probiotics supplementation on aerobic and anaerobic performance in athletes. Not all of these processes are well understood, and there is a clear need for future studies to explore this intestine-muscle connection. These studies should be focused on athletes and strive to enhance our understanding of their physiological muscle function maintenance.

## Figures and Tables

**Table 1:** The effect of microbiota on exercise.

References	Study Model	Type of Exercise	Intervention	Beneficial Effect of Intervention on Direct and Indirect Parameters of Sports Performance
Hsu et al. 2015 [119]	Mice	Endurance swimming	**Threestudy groups:** germ free (GF) vs. *Bacteroides fragilis* (BF) comparison with no probiotic (specific pathogen-free (SPF)	↑ activity of serum glutathione peroxidase (GPx) and catalase (Cat) ↑ activity of liver GPx ↑ muscle mass ↑ antioxidant properties ↑ free radical damage protection ↑ muscle mass endurance (extended exercise to exhaustion time) No differences in liver superoxide dismutase (SOD) and Cat activity
Unsal et al. 2018 [120]	Rats	Exhaustive swimming trial	**Fourstudy groups**: control, placebo, exercise, exercise+probiotic	↓ oxidative stress ↑ antioxidative enzymes activity ↑ antioxidative balance
			**Study product**: multi strain probiotic mixture VSL#3 (*Lactobacillus casei, L. plantarum, L. acidophilus, L. delbrueckii subsp. bulgaricus, Bifidobacterium longum, B. breve,* and *B. infantis, Streptococcus salivarius*)	
Scheiman et al. 2019 [97]	Mice	Exhaustive treadmill run	**Twostudy groups:** control and supplemented	↑ lactate utilization ↑ blood short-chain fatty acid (SCFA) concentration ↑ extended exercise to exhaustion time (treadmill workout) ↑ Cori cycle efficiency
			**Studyproduct**: *Veilonella*, propionic	
Chen et al. 2016 [52]	Mice	grip strength and endurance swimming	**Threestudy groups:** vehicle, 2.05ˆ108CFU/kg (LP10-1X), and 1.03ˆ109CFU/kg (LP10-5X).	↑ relative muscle mass and strength ↑ number of type 1 muscle fibers ↑ extended exercise to exhaustion time (swimming trial) ↓ post-workout lactate blood concentration ↓ post-workout ammonia blood concentration ↓ post-workout CK ↓ post-workout ammonia, albumin, creatinine and triglyceride concentration All above changes were dose-dependent
			**Study product**: *Lactobacillus plantarum* TWK10 (LP10)	
Hoffman et al. 2019 [123]	Soldiers	vertical jump power, two times 100-m shuttle runs	**Twostudy groups**: *Bacillus coagulans* and placebo	↑ interferon gamma (IFN)-γ and interleukin-10 (IL-10) concentration ↑ mean jump power No effects on 60 s pull-ups, 100-m shuttle run, shuttle run fatigue rate No effects on cortisol and testosterone concentration No effects on CK and pro-inflammatory cytokines concentration
			**Studyproduct:** *Bacillus coagulans*	
Jager et al. 2016 [121]	Recreative training man	Damaging exercise bout	**Twostudy groups**: 20 g of casein consumptionand/or 20 g of casein plus *Bacillus* consumption	↑ regeneration perception after damaging workout ↑ sport performance in Wingate Test ↓ soreness perception 24 and 72 h after damaging workout ↓ post-exercise blood CK No effects on muscle strength and thickness
			**Study product**: *Bacillus coagulans* GBI-30	
Roberts et al. 2016 [81]	untrained men and women	triathlon specific stage times (swim, bike, and run)	**Three study gorups:** probiotics, probiotics +antioxidants and placebo	↓ blood lipopolysaccharide (LPS) level up to 6 days after workout ↓ race duration
			**Study product:** mix of *Bifidobaterium* and *Lactobacillus*	
Toohey et.al. 2018 [124]	volleyballplayers (women)	squat, deadlift, and bench press, vertical jump, pro-agility and isometric midthigh pull test	**Twostudy groups:** probiotic and placebo	↓ fat mass level compared to placebo group No effects on strength or athletic performance.
			**Studyproduct:** *Bacillus Subtilis*	
Jager et al. 2016 [78]	resistance-trained men	eccentric exercise of the elbow	**Twostudy groups:** probiotic and placebo	↓ IL-6 concentration up 48 h after damaging training ↑ maximal voluntary isometric peak torque at 24 to 72 h following damaging exercises ↑ flexed arm angle after damaging workout No effect on average maximal voluntary isometric peak No clear effect on plasma CK level after damaging exercises
			**Study product:***Streptococcus thermophilus* FP4 *Bifidobacterium breve* BR03	
Carbuhn et al. 2018 [122]	Swimmers (women)	aerobic/anaerobic swim time trials and force plate vertical jump	**Twostudy groups**: probiotic and placebo	↑ post-training regeneration perception No effects on aerobic and anaerobic swim performance testing No effects onconcentric/eccentric force production No differences in serum IL-1, LPS, and LPS Binding Protein (LBP) concentration
			**Studyproduct:***Bifidobacterium longum* 35624	
Townsend et al. 2018 [80]	baseball players (men)	Ten-yard sprint test, pro-agility test, standing long jump	**Twogroups**: probiotics and placebo	↓ post-workout blood TNF-α concentration No significant effecton IL-10, zonulin, testosterone, cortisol concentration and salivary immunoglobulin A (SIgA) secretion No differences in strength, performance and body composition
			**Studyproduct**: *Bacillus subtilis* DE111	
Huang et al. 2019 [79]	triathletes	triathlon championship	**Twostudy groups**: *Lactobacillus* and placebo	↓ oxidative stress level ↑ antioxidant potential through thioredoxin (TRX) and MPO modulation ↑ post-workout blood BCAA concentration ↑ post-workout regeneration rate ↑ post-workout blood IL-10 concentration ↓ post-workout blood IL-6, IL-8, TNF-α IFN-γ concentration ↓ CK level during recovery period ↑ anaerobic capacity in Wingate Test No significant differences in body composition No effects on CK, myoglobin, lactate dehydrogenase (LDH), ammonia, lactate and FFA after exercise
			**Study product**: *Lactobacillus plantarum* PS128	

Table symbols: ↑—increase; ↓—decrease.

## References

[1] Thursby E., Juge N. (2017). Introduction to the human gut microbiota. Biochem. J..

[2] Gary D.W., Bushmanc F.D., Lewis J.D. (2013). Diet, the human gut microbiota, and IBD. Anaerobe.

[3] Shreiner A.B., Kao J.Y., Young V.B. (2015). The gut microbiome in health and in disease. Curr. Opin. Gastroenterol.

[4] Mach N., Fuster-Botella D. (2017). Endurance exercise and gut microbiota: A review. J. Sport Health Sci..

[5] Rothschild D., Weissbrod O., Barkan E., Kurilshikov A., Korem T., Zeevi D. (2018). Environment dominates over host genetics in shaping human gut microbiota. Nature.

[6] Vich Vila A., Collij V., Sanna S., Sinha T., Imhann F., Bourgonje A.R. (2020). Impact of commonly used drugs on the composition and metabolic function of the gut microbiota. Nat. Commun..

[7] Das B., Nair G.B. (2019). Homeostasis and dysbiosis of the gut microbiome in health and disease. J. Biosci..

[8] Hearris M.A., Hammond K.M., Fell J.M., Morton J.P. (2018). Regulation of Muscle Glycogen Metabolism during Exercise: Implications for Endurance Performance and Training Adaptations. Nutrients.

[9] Atherton P.J., Smith K. (2012). Muscle protein synthesis in response to nutrition and exercise. J. Physiol..

[10] McCarthy J.J., Esser K.A. (2010). Anabolic and catabolic pathways regulating skeletal muscle mass. Curr. Opin. Clin. Nutr. Metab. Care.

[11] Ji L.L., Gomez-Cabrera M.C., Steinhafel N., Vina J. (2004). Acute exercise activates nuclear factor (NF)-κB signaling pathway in rat skeletal muscle. FASEB J..

[12] Sohail M.U., Yassine H.M., Sohail A., Al Thani A.A. (2019). Impact of Physical Exercise on Gut Microbiome, Inflammation, and the Pathobiology of Metabolic Disorders. Rev. Diabet. Stud..

[13] Coleman N. (2019). Gastrointestinal Issues in Athletes. Curr. Sports Med. Rep..

[14] De Kivit S., Tobin M.C., Forsyth C.B., Keshavarzian A., Landay A.L. (2014). Regulation of Intestinal Immune Responses through TLR Activation: Implications for Pro- and Prebiotics. Front. Immunol..

[15] McCall L.M., Ackerman K.E. (2019). Endocrine and metabolic repercussions of relative energy deficiency in sport. Curr. Opin. Endocr. Metab. Res..

[16] Mountjoy M., Sundgot-Borgen J., Burke L., Carter S., Constantini N., Lebrun C. (2014). The IOC consensus statement: Beyond the Female Athlete Triad—Relative Energy Deficiency in Sport (RED-S). Br. J. Sports Med..

[17] Spriet L.L. (2014). New Insights into the Interaction of Carbohydrate and Fat Metabolism during Exercise. Sports Med..

[18] Philp A., Hargreaves M., Baar K. (2012). More than a store: Regulatory roles for glycogen in skeletal muscle adaptation to exercise. Am. J. Physiol. Endocrinol. Metab..

[19] Gentile C.L., Weir T.L. (2018). The gut microbiota at the intersection of diet and human health. Science.

[20] Shimizu H., Masujima Y., Ushiroda C., Mizushima R., Taira S., Ohue-Kitano R. (2019). Dietary short-chain fatty acid intake improves the hepatic metabolic condition via FFAR3. Sci. Rep..

[21] Burke L.M., Ross M.L., Garvican-Lewis L.A., Welvaert M., Heikura I.A., Forbes S.G. (2017). Low carbohydrate, high fat diet impairs exercise economy and negates the performance benefit from intensified training in elite race walkers. J. Physiol..

[22] Crawford M., Whisner C., Al-Nakkash L., Sweazea K.L. (2019). Six-Week High-Fat Diet Alters the Gut Microbiome and Promotes Cecal Inflammation, Endotoxin Production, and Simple Steatosis without Obesity in Male Rats. Lipids..

[23] Wu G.D., Chen J., Hoffmann C., Bittinger K.Y., Chen Y., Keilbaugh S.A. (2011). Linking Long-Term Dietary Patterns with Gut Microbial Enterotypes. Science.

[24] Rinninella E., Cintoni M., Raoul P., Lopetuso L.R., Scaldaferri F., Pulcini G. (2019). Food Components and Dietary Habits: Keys for a Healthy Gut Microbiota Composition. Nutrients.

[25] Den Hartigh L.J. (2019). Conjugated Linoleic Acid Effects on Cancer, Obesity, and Atherosclerosis: A Review of Pre-Clinical and Human Trials with Current Perspectives. Nutrients.

[26] Churchward-Venne T.A., Burd N.A., Mitchell C.J., West D.W.D., Philp A., Marcotte G.R. (2012). Supplementation of a suboptimal protein dose with leucine or essential amino acids: Effects on myofibrillar protein synthesis at rest and following resistance exercise in men. J. Physiol..

[27] Dallas D.C., Sanctuary M.R., Qu Y., Khajavi S.H., van Zandt A.E., Dyandra M. (2017). Personalizing protein nourishment. Crit. Rev. Food Sci. Nutr..

[28] Chassard C., Lacroix C. (2013). Carbohydrates and the human gut microbiota. Curr. Opin. Clin. Nutr. Metab. Care.

[29] Wu G.D., Compher C., Chen E.Z., Smith S.A., Shah R.D., Bittinger K. (2016). Comparative metabolomics in vegans and omnivores reveal constraints on diet-dependent gut microbiota metabolite production. Gut.

[30] Kårlund A., Gómez-Gallego C., Turpeinen A.M., Palo-Oja O.M., El-Nezami H., Kolehmainen M. (2019). Protein Supplements and Their Relation with Nutrition, Microbiota Composition and Health: Is More Protein Always Better for Sportspeople?. Nutrients.

[31] Mika A., van Treuren W., González A., Herrera J.J., Knight R., Fleshner M. (2015). Exercise is More Effective at Altering Gut Microbial Composition and Producing Stable Changes in Lean Mass in Juvenile versus Adult Male F344 Rats. PLoS ONE.

[32] Barton W., Penney N.C., Cronin O., Garcia-Perez I., Molloy M.G., Holmes E. (2018). The microbiome of professional athletes differs from that of more sedentary subjects in composition and particularly at the functional metabolic level. Gut..

[33] Petersen L.M., Bautista E.J., Nguyen H., Hanson B.M., Chen L., Lek S.H. (2017). Community characteristics of the gut microbiomes of competitive cyclists. Microbiome.

[34] Durk R.P., Castillo E., Márquez-Magaña L., Grosicki G.J., Bolter N.D., Lee C.M. (2019). Gut Microbiota Composition Is Related to Cardiorespiratory Fitness in Healthy Young Adults. Int. J. Sport Nutr. Exerc. Metab..

[35] Małkiewicz M.A., Szarmach A., Sabisz A., Cubała W.J., Szurowska E., Winklewski P.J. (2019). Blood-brain barrier permeability and physical exercise. J. Neuroinflammation.

[36] Cervenka I., Agudelo L.Z., Ruas J.L. (2017). Kynurenines: Tryptophan’s metabolites in exercise, inflammation, and mental health. Science.

[37] Karl J.P., Margolis L.M., Madslien E.H., Murphy N.E., Castellani J.W., Gundersen Y. (2017). Changes in intestinal microbiota composition and metabolism coincide with increased intestinal permeability in young adults under prolonged physiological stress. Am. J. Physiol. Gastrointest. Liver Physiol..

[38] Peternelj T.T., Coombes J.S. (2011). Antioxidant Supplementation during Exercise Training. Sports Med..

[39] Safdar A., Hamadeh M.J., Kaczor J.J., Raha S., Debeer J., Tarnopolsky M.A. (2010). Aberrant Mitochondrial Homeostasis in the Skeletal Muscle of Sedentary Older Adults. PLoS ONE.

[40] Kaczor J.J., Robertshaw H.A., Tarnopolsky M.A. (2017). Higher Oxidatove Stress in Skeletal Muscle of McArdle Disease Patients. Mol. Genet. Metab. Rep..

[41] Pingitore A., Lima G.P.P., Mastorci F., Quinones A., Iervasi G., Vassalle C. (2015). Exercise and oxidative stress: Potential effects of antioxidant dietary strategies in sports. Nutrition.

[42] HajizadehMaleki B., Tartibian B., Eghbali M., Asri-Rezaei S. (2013). Comparison of seminal oxidants and antioxidants in subjects with different levels of physical fitness. Andrology.

[43] Brinkmann C., Chung N., Schmidt U., Kreutz T., Lenzen E., Schiffer T. (2012). Training alters the skeletal muscle antioxidative capacity in non-insulin-dependent type 2 diabetic men. Scand. J. Med. Sci. Sports.

[44] Brandt N., Gunnarsson T.P., Hostrup M., Tybirk J., Nybo L., Pilegaard H. (2016). Impact of adrenaline and metabolic stress on exercise-induced intracellular signaling and PGC-1α mRNA response in human skeletal muscle. Physiol. Rep..

[45] Schoenfeld B.J. (2012). Does exercise-induced muscle damage play a role in skeletal muscle hypertrophy?. J. Strength Cond. Res..

[46] Martarelli D., Verdenelli M.C., Scuri S., Cocchioni M., Silvi S., Cecchini C. (2011). Effect of a probiotic intake on oxidant and antioxidant parameters in plasma of athletes during intense exercise training. Curr. Microbiol..

[47] Spyropoulos B.G., Misiakos E.P., Fotiadis C., Stoidis C.N. (2011). Antioxidant properties of probiotics and their protective effects in the pathogenesis of radiation-induced enteritis and colitis. Dig. Dis. Sci..

[48] Qiao Y., Sun J., Ding Y., Le G., Shi Y. (2013). Alterations of the gut microbiota in high-fat diet mice is strongly linked to oxidative stress. Appl. Microbiol. Biotechnol..

[49] Ticinesi A., Lauretani F., Tana C., Nouvenne A., Ridolo E., Meschi T. (2019). Exercise and immune system as modulators of intestinal microbiome: Implications for the gut-muscle axis hypothesis. Exerc. Immunol. Rev..

[50] Ni Lochlainn M., Bowyer R.C.E., Steves C.J. (2018). Dietary Protein and Muscle in Aging People: The Potential Role of the Gut Microbiome. Nutrients.

[51] Bindels L.B., Beck R., Schakman O., Martin J.C., De Backer F., Sohet F.M. (2012). Restoring Specific Lactobacilli Levels Decreases Inflammation and Muscle Atrophy Markers in an Acute Leukemia Mouse Model. PLoS ONE.

[52] Chen Y.M., Wei L., Chiu Y.S., Hsu Y.J., Tsai T.Y., Wang M.F. (2016). Lactobacillus plantarum TWK10 Supplementation Improves Exercise Performance and Increases Muscle Mass in Mice. Nutrients.

[53] Buigues C., Fernández-Garrido J., Pruimboom L., Hoogland A.J., Navarro-Martínez R., Martínez-Martínez M. (2016). Effect of a Prebiotic Formulation on Frailty Syndrome: A Randomized, Double-Blind Clinical Trial. Int. J. Mol. Sci..

[54] Xiao S., Fei N., Pang X., Shen J., Wang L., Zhang B. (2014). A gut microbiota-targeted dietary intervention for amelioration of chronic inflammation underlying metabolic syndrome. FEMS Microbiol. Ecol..

[55] Ahola A.J., Lassenius M.I., Forsblom C., Harjutsalo V., Lehto M., Groop P.H. (2017). Dietary patterns reflecting healthy food choices are associated with lower serum LPS activity. Sci. Rep..

[56] Salguero M., Al Obaide M., Singh R., Siepmann T., Vasylyeva T. (2019). Dysbiosis of Gram-negative gut microbiota and the associated serum lipopolysaccharide exacerbates inflammation in type 2 diabetic patients with chronic kidney disease. Exper. Ther. Med..

[57] Stevens B.R., Goel R., Seungbum K., Richards E.M., Holbert R.C., Pepine C.J. (2018). Increased human intestinal barrier permeability plasma biomarkers zonulin and FABP2 correlated with plasma LPS and altered gut microbiome in anxiety or depression. Gut.

[58] Lamprecht M., Frauwallner A. (2012). Exercise, intestinal barrier dysfunction and probiotic supplementation. Med. Sport Sci..

[59] Schirmer M., Smeekens S.P., Vlamakis H., Jaeger M., Oosting M., Franzosa E.A. (2016). Linking the Human Gut Microbiome to Inflammatory Cytokine Production Capacity. Cell.

[60] Linsalata M., Riezzo G., D’Attoma B., Clemente C., Orlando A., Russo F. (2018). Noninvasive biomarkers of gut barrier function identify two subtypes of patients suffering from diarrhoea predominant-IBS: A case-control study. BMC Gastroenterol..

[61] Konturek P.C., Brzozowski T., Konturek S.J. (2011). Stress and the gut: Pathophysiology, clinical consequences, diagnostic approach and treatment options. J. Physiol. Pharmacol..

[62] Round J.L., Lee S.M., Li J., Tran G., Jabri B., Chatila T.A. (2011). The Toll-like receptor 2 pathway establishes colonization by a commensal of the human microbiota. Science.

[63] Strzępa A., Szczepanik M. (2013). Influence of natural gut flora on immune response. Postepy Hig. Med. Dosw..

[64] McCarthy J.J., Mula J., Miyazaki M., Erfani R., Garrison K., Farooqui A.B. (2011). Effective fiber hypertrophy in satellite cell-depleted skeletal muscle. Development.

[65] Gumucio J.P., Mendias C.L. (2013). Atrogin-1, MuRF-1, and sarcopenia. Endocrine.

[66] Liu H.W., Chen Y.J., Chang Y.C., Chang S.J. (2017). Oligonol, a Low-Molecular Weight Polyphenol Derived from Lychee, Alleviates Muscle Loss in Diabetes by Suppressing Atrogin-1 and MuRF1. Nutrients.

[67] Bonaldo P., Sandri M. (2013). Cellular and molecular mechanisms of muscle atrophy. Dis. Model. Mech..

[68] Barclay R.D., Burd N.A., Tyler C., Tillin N.A., Mackenzie R.W. (2019). The Role of the IGF-1 Signaling Cascade in Muscle Protein Synthesis and Anabolic Resistance in Aging Skeletal Muscle. Front. Nutr..

[69] Philippou A., Barton E.R. (2014). Optimizing IGF-I for skeletal muscle therapeutics. Growth Horm. IGF Res..

[70] Sanchez A.M., Candau R., Bernardi H. (2019). Recent Data on Cellular Component Turnover: Focus on Adaptations to Physical Exercise. Cells.

[71] Sanchez A.M.J., Csibi A., Raibon A., Cornille K., Gay S., Bernardi H. (2012). AMPK promotes skeletal muscle autophagy through activation of forkhead FoxO3a and interaction with Ulk1. J. Cell Biochem..

[72] Sanchez A.M.J., Bernardi H., Py G., Candau R.B. (2014). Autophagy is essential to support skeletal muscle plasticity in response to endurance exercise. Am. J. Physiol. Regul. Integr. Comp. Physiol..

[73] da Rocha A.L., Pinto A.P., Kohama E.B., Pauli J.R., de Moura L.P., Cintra D.E. (2019). The proinflammatory effects of chronic excessive exercise. Cytokine.

[74] Shephard R.J., Shek P.N. (1998). Acute and chronic over-exertion: Do depressed immune responses provide useful markers?. Int. J. Sports Med..

[75] Angeli A., Minetto M., Dovio A., Paccotti P. (2004). The overtraining syndrome in athletes: A stress-related disorder. J. Endocrinol. Investig..

[76] Smith L.L. (2000). Cytokine hypothesis of overtraining: A physiological adaptation to excessive stress?. Med. Sci. Sports Exerc..

[77] Borges L.S., Dermargos A., da Silva Junior E.P., Weimann E., Lambertucci R.H., Hatanaka E. (2015). Melatonin decreases muscular oxidative stress and inflammation induced by strenuous exercise and stimulates growth factor synthesis. J. Pineal. Res..

[78] Jäger R., Purpura M., Stone J.D., Turner S.M., Anzalone A.J., Eimerbrink M.J. (2016). Probiotic Streptococcus thermophilus FP4 and Bifidobacteriumbreve BR03 Supplementation Attenuates Performance and Range-of-Motion Decrements Following Muscle Damaging Exercise. Nutrients.

[79] Huang W.C., Wei C.C., Huang C.C., Chen W.L., Huang H.Y. (2019). The Beneficial Effects of Lactobacillus plantarum PS128 on High-Intensity, Exercise-Induced Oxidative Stress, Inflammation, and Performance in Triathletes. Nutrients.

[80] Townsend J., Bender D., Vantrease W., Sapp P., Toy A., Woods C. (2018). Effects of Probiotic (Bacillus subtilis DE111) Supplementation on Immune Function, Hormonal Status, and Physical Performance in Division I Baseball Players. Sports.

[81] Roberts J.D., Suckling C.A., Peedle G.Y., Murphy J.A., Dawkins T.G., Roberts M.G. (2016). An Exploratory Investigation of Endotoxin Levels in Novice Long Distance Triathletes, and the Effects of a Multi-Strain Probiotic/Prebiotic, Antioxidant Intervention. Nutrients.

[82] Marchesi J.R., Adams D.H., Fava F., Hermes G.D.A., Hirschfield G.M., Hold G. (2016). The gut microbiota and host health: A new clinical frontier. Gut.

[83] Macfarlane G.T., Macfarlane S. (2011). Fermentation in the human large intestine: Its physiologic consequences and the potential contribution of prebiotics. J. Clin. Gastroenterol..

[84] Magnusson M.K., Isaksson S., Öhman L. (2019). The Anti-inflammatory Immune Regulation Induced by Butyrate Is Impaired in Inflamed Intestinal Mucosa from Patients with Ulcerative Colitis. Inflammation.

[85] Hernández M.A.G., Canfora E.E., Jocken J.W.E., Blaak E.E. (2019). The Short-Chain Fatty Acid Acetate in Body Weight Control and Insulin Sensitivity. Nutrients.

[86] Kasubuchi M., Hasegawa S., Hiramatsu T., Ichimura A., Kimura I. (2015). Dietary gut microbial metabolites, short-chain fatty acids, and host metabolic regulation. Nutrients.

[87] Samuel B.S., Shaito A., Motoike T., Rey F.E., Backhed F., Manchester J.K., Hammer R.E., Williams S.C., Crowley J., Yanagisawa M. (2008). Effects of the gut microbiota on host adiposity are modulated by the short-chain fatty-acid binding G protein-coupled receptor, Gpr41. Proc. Natl. Acad. Sci. USA.

[88] den Besten G., van Eunen K., Groen A.K., Venema K., Reijngoud D.J. (2013). The role of short-chain fatty acids in the interplay between diet, gut microbiota, and host energy metabolism. J. Lipid. Res..

[89] Cerdá B., Pérez M., Pérez-Santiago J.D., Tornero-Aguilera J.F., González-Soltero R., Larrosa M. (2016). Gut Microbiota Modification: Another Piece in the Puzzle of the Benefits of Physical Exercise in Health?. Front. Physiol..

[90] Kobayashi Y., Hara N., Sugimoto R., Mifuji-Moroka R., Tanaka H., Eguchi A. (2017). The Associations between Circulating Bile Acids and the Muscle Volume in Patients with Non-alcoholic Fatty Liver Disease (NAFLD). Intern. Med..

[91] Sayin S.I., Wahlström A., Felin J., Jäntti S., Marschall H.U., Bamberg K. (2013). Gut microbiota regulates bile acid metabolism by reducing the levels of tauro-beta-muricholic acid, a naturally occurring FXR antagonist. Cell Metab..

[92] Lin R., Liu W., Piao M., Zhu H. (2017). A review of the relationship between the gut microbiota and amino acid metabolism. Amino Acids.

[93] Neis E., Dejong C., Rensen S. (2015). The Role of Microbial Amino Acid Metabolism in Host Metabolism. Nutrients.

[94] LeBlanc J.G., Milani C., de Giori G.S., Sesma F., van Sinderen D., Ventura M. (2013). Bacteria as vitamin suppliers to their host: A gut microbiota perspective. Curr. Opin. Biotechnol..

[95] Pereira-Caro G., Polyviou T., Ludwig I.A., Nastase A.M., Moreno-Rojas J.M., Garcia A.L. (2017). Bioavailability of orange juice (poly)phenols: The impact of short-term cessation of training by male endurance athletes. Am. J. Clin. Nutr..

[96] Kang C.Y., Halabi W.J., Luo R., Pigazzi A., Nguyen N.T., Stamos M.J. (2012). Laparoscopic colorectal surgery: A better look into the latest trends. Archiv. Surg..

[97] Scheiman J., Luber J.M., Chavkin T.A., MacDonald T., Tung A., Pham L.D. (2019). Meta-omics analysis of elite athletes identifies a performance-enhancing microbe that functions via lactate metabolism. Nat. Med..

[98] Chambers E.S., Byrne C.S., Aspey K., Chen Y., Khan S., Morrison D.J. (2018). Acute oral sodium propionate supplementation raises resting energy expenditure and lipid oxidation in fasted humans. Diabetes Obes. Metab..

[99] Kimura I., Inoue D., Maeda T., Hara T., Ichimura A., Miyauch S. (2011). Short-chain fatty acids and ketones directly regulate sympathetic nervous system via G protein-coupled receptor 41 (GPR41). Proc. Natl. Natl. Sci. USA.

[100] Pokrzywnicka P., Gumprecht J. (2017). Intestinal microbiota and its relationship with diabetes and obesity. Clin. Diabetol..

[101] Nay K., Jollet M., Goustard B., Baati N., Vernus B., Pontones M. (2019). Gut bacteria are critical for optimal muscle function: A potential link with glucose homeostasis. Am. J. Physiol. Endocrinol. Metab..

[102] Zarrinpar A., Chaix A., Xu Z.Z., Chang M.W., Marotz C.A., Saghatelian A. (2018). Antibiotic-induced microbiome depletion alters metabolic homeostasis by affecting gut signaling and colonic metabolism. Nat. Commun..

[103] Saint-Georges-Chaumet Y., Edeas M. (2016). Microbiota–mitochondria inter-talk: Consequence for microbiota–host interaction. Pathog. Dis..

[104] Lebreton A., Stavru F., Cossart P. (2015). Organelle targeting during bacterial infection: Insights from Listeria. Trends Cell Biol..

[105] Donohoe D.R., Garge N., Zhang X., Sun W., O’Connell T.M., Bunger M.K. (2011). The microbiome and butyrate regulate energy metabolism and autophagy in the mammalian colon. Cell Metab..

[106] Houghton M.J., Kerimi A., Mouly V., Tumova S., Williamson G. (2019). Gut microbiomecatabolites as novel modulators of muscle cell glucose metabolism. FASEB J..

[107] Li M.E., Lauritzen H.P.M.M., O’Neill B.T., Wang C.-H., Cai W., Brandao B.B. (2019). Role of p110a subunit of PI3-kinase in skeletal muscle mitochondrial homeostasis and metabolism. Nat. Commun..

[108] Baj A., Moro E., Bistoletti M., Orlandi V., Crema F., Giaroni C. (2019). Glutamatergis Signaling Along The Mivrobiota-Gut-Brain Axis. Int. J. Mol. Sci..

[109] Furness J.B., Callaghan B.P., Rivera L.R., Cho H.-J. (2014). The enteric nervous system and gastrointestinal innervation: Integrated local and central control. Adv. Exp. Med. Biol..

[110] Bermon S., Petriz S., Kajėnienė A., Prestes J., Castell L., Franco O.L. (2015). The microbiota: An exercise immunology perspective. Exerc. Immunol. Rev..

[111] Clarke G., Stilling R.M., Kennedy P.J., Stanton C., Cryan J.F., Dinan T.G. (2014). Minireview: Gut Microbiota: The Neglected Endocrine Organ. Mol. Endocrinol..

[112] Clark A., Mach N. (2016). Exercise-induced stress behavior, gutmicrobiota-brain axis and diet: A systematicreview for athletes. J. Int. Soc. Sports Nutr..

[113] Bravo J.A., Forsythe P., Chew M.V., Escaravage E., Savignac H.M., Dinan T.G. (2011). Ingestion of *Lactobacillus* strain regulates emotional behavior and central GABA receptor expression in the Mouse via the vagus nerve. Proc. Natl. Acad. Sci. USA.

[114] Strandwitz P. (2018). Neurotransmitter modulation by the gut microbiota. Brain Res..

[115] Crumeyrolle-Arias M., Jaglin M., Bruneau A., Vancassel S., Cardona A., Dauge V. (2014). Absence of the Gut Microbiota Enhances Anxiety-Like Behavior and Neuroendocrine Response to Acute Stress in Rats. Psychoneuroendocrinology..

[116] Karnia M.J., Myslińska D., Dzik K.P., Flis D.J., Ciepielewski Z.M., Podlacha M., Kaczor J.J. (2018). The Electrical Stimulation of the Bed Nucleus of the StriaTerminalis Causes Oxidative Stress in Skeletal Muscle of Rats. Oxid. Med. Cell Longev..

[117] Karnia M.J., Myślińska D., Dzik K.P., Flis D.J., Podlacha M., Kaczor J.J. (2020). BST Stimulation Induces Atrophy and Changes in Aerobic Energy Metabolism in Rat Skeletal Muscles-The Biphasic Action of Endogenous Glucocorticoids. Int. J. Mol. Sci..

[118] Kuo T., Harris C.A., Wang J.C. (2013). Metabolic functions of glucocorticoid receptorin skeletal muscle. Mol. Cell. Endocrinol..

[119] Hsu Y.J., Chiu C.C., Li Y.P., Huang W.C., Huang Y.T., Huang C.C. (2015). Effect of intestinal microbiota on exercise performance in mice. J. Strength Cond. Res..

[120] Ünsal C., Ünsal H., Ekici M., KoçYildirim E., Üner A.G., Yildiz M. (2018). The effects of exhaustive swimming and probiotic administration in trained rats: Oxidative balance of selected organs, colon morphology, and contractility. Physiol. Int..

[121] Jäger R., Shields K.A., Lowery R.P., De Souza E.O., Partl J.M., Hollmer C. (2016). Probiotic Bacillus coagulans GBI-30, 6086 reduces exercise-induced muscle damage and increases recovery. Peer. J..

[122] Carbuhn A., Reynolds S., Campbell C., Bradford L., Deckert J., Kreutzer A. (2018). Effects of Probiotic (Bifidobacteriumlongum 35624) Supplementation on Exercise Performance, Immune Modulation, and Cognitive Outlook in Division I Female Swimmers. Sports.

[123] Hoffman J.R., Hoffman M.W., Zelicha H., Gepner Y., Willoughby D.S., Feinstein U. (2019). The Effect of 2 Weeks of Inactivated Probiotic Bacillus coagulans on Endocrine, Inflammatory, and Performance Responses During Self-Defense Training in Soldiers. J. Strength Cond. Res..

[124] Toohey J.C., Townsend J.R., Johnson S.B., Toy A.M., Vantrease W.C., Bender D. (2018). Effects of Probiotic (Bacillus subtilis) Supplementation During Offseason Resistance Training in Female Division I Athletes. J. Strength Cond. Res..

